# Report on Cases of Fever Occurring in Guy’s Hospital, in 1854

**Published:** 1856-03

**Authors:** Samuel Wilks


*Report on all the cases of Fever which occurred in Guy's Hospital during the year* 1854, *with Remarks having especial reference to the Typhus and Typhoid distinctions.* By SAMUEL WILKS, M. D. (Guy's Hospital Reports, 1855.)

The question at present which excites most interest in connection with fever, is the identity or non-identity of typhus and typhoid fever. This question is, no doubt, set at rest in the minds of many. It is set at rest in our own mind, and we fully agree with Dr. Jenner in thinking that typhus and typhoid are different affections. But this is not the case with all, and therefore we are glad to have the additional evidence which is contained in the present report.

This evidence, then, consists of the notes of no less than 187 cases. It is clear and conclusive; and, after reading it, it is scarcely possible that any unprejudiced person should continue to believe in the identity of these two forms of fever. We cannot give the cases; we can only give some of the comments upon them.

"It will be seen," says Dr. Wilks, "that two very different rashes are described, and that these have occurred in various degrees of intensity, and that they have sometimes been altogether absent. In the latter cases, it will be observed, however, that with the exception of the eruption, the symptoms in every respect followed a particular type. Regarding, first of all, the cases which had the rashes, it will be seen that those in which the rose eruption existed had, without exception, diarrhœa as well as other symptoms of intestinal disease, and, in fact, they were all well-marked cases of typhoid fever. Those with a mulberry rash had for the most part a comparatively healthy state of bowels, although it is mentioned that diarrhœa occurred in one or two instances, but the importance of this symptom we shall presently discuss. As regards the relation of the degree of the virulence of the fever to the amount of the rash, there is not much to be learned, as it will be seen that the violence of the former, and the degree of the intensity of the latter, bore no exact proportion to each other; although it is true that the mildest cases had no rash, and that in the most severe it was generally present, and also that no fatal case in the above list occurred when it was altogether wanting. (I have seen typhoid fever fatal, however, without any eruption, although I have never witnessed this occurrence in typhus.) The rash argues, therefore, a certain amount of intensity of the fever, but is not proportionate to it. Its presence in all the fatal cases proves so much. Eliminating, however, the extreme instances, and regarding only the majority of the ordinary cases, we cannot say that the amount of the rash, and the severity of the disease, as shown by other symptoms, were proportionate. It will be seen that in some cases, as No. 73, where the mulberry rash was well developed, the symptoms were, comparatively speaking, mild, and the patient recovered without the administration of any stimulant. On the other hand, No. 90 is an example of a man who had no eruption, and yet who narrowly escaped death. The same remarks hold good of the rose rash. I have more than once seen out-patients present themselves at the hospital with fever, in whom a rose rash existed, and the accompanying symptoms were of the mildest form. In case 144, if the eruption had not been present, the existence of fever would hardly have been determined; and the patient was so little ill, that his principal trouble was the order for keeping his bed. It is clear, then, that there are other causes besides the mere virulence of the disease, which determine the presence and amount of the eruption, and I am inclined to think that these depend upon accidental causes, as some peculiar idiosyncrasy of the patient, which predisposes him to affections of the skin, or upon some condition of the integument itself, which makes it more liable to assume a morbid state in one person than another—in the same way as some are prone to have pulmonic, and others to have cerebral complication when attacked with fever. I would be understood to say, that the presence and peculiar character of the rash is primarily determined by the specific agency in operation, and that, to a certain extent, the more powerful that agency the greater is the tendency to the skin affection, as evidenced by its existence in fatal cases, but that after the peculiar rash has been so produced, the fact that it does not vary in intensity with that of the fever, proves that some other conditions are present to influence its development, and these, I cannot but think, are mostly accidental, and due to the nature of the skin itself, or some such secondary cause. In the analogous instance of scarlatina, we see the mildest and the worst cases where little or no eruption exists, so that no relation can be determined between the virulence of the disease and the amount of the rash.

"Now with reference to the two varieties of eruption—the typhus and the typhoid—there is, as a rule, a maiked difference between them. The clear skin with the pink spots scattered over it in the latter, and the mottled skin of the former, are generally sufficiently distinct. How the two can be confounded, in the majority of instances, I cannot well imagine, and yet some writers at the present time have not perceived a difference. I think this must arise from a want of extensive observation, for no doubt occasionally, in particular instances, great difficulties do arise. Thus, we sometimes see patients exceedingly low, the whole surface of the skin dusky, perhaps also very dirty, and covered with fleabites, and, in addition, numerous acne and other pimples; and amongst all this is to be observed a recent rash, which fades on pressure. In some such cases I have found it almost impossible to say whether these were the rose spots of typhoid, or the larger marks of the mulberry of typhus. Such instances have appeared to favor the opinion that no distinction existed between them; that the two rashes were mere modifications of one another; or, if distinct, that they could occur at the same time, and consequently, that the two forms of fever were undistinguishable. If the rash alone were to determine the whole question of the generic differences of fever, such cases would no doubt often warrant such a conclusion; but the whole history of the case must be taken to discover its nature. For example, No. 127 was that of a woman, who, on entering the hospital, was too ill to give any account of herself. The whole body was covered with spots, ill defined, and the skin was dusky, and it was difficult to say to which kind the eruption belonged. Thus she remained two days, when the cheeks became flushed, the bowels very loose, and a friend who now arrived stated the duration of the illness, and the previous existence of diarrhœa. At the same time the spots became more characteristic, and the case run a uniform one of typhoid. In this instance, with the absence of history, and no very marked symptoms, the eruption would altogether have failed to determine the nature of the case at the time of admission. In its subsequent course, however, as well as in its previous history, it showed itself to be one of ordinary typhoid, and, therefore, teaches us that the obscurity of the rash alone cannot warrant us in the assertion that typhus and typhoid are inextricably united. In two other instances, also, there was a difference of opinion among good observers as to which kind the rash was to be referred. These are, however, exceptional cases, for in the majority it must be affirmed that the two eruptions are well marked, are characteristic, and cannot be confounded. It has been farther questioned, even allowing the existence of a general distinction between them, whether they constitute more than varieties of each other, dependent upon secondary circumstances, and whether the two may not in some instances be combined. In such cases as I have just mentioned, it would be difficult to answer such a query positively or negatively; for amongst a thick typhoid rash it would be impossible to say that some spots did not remain during the whole time of the fever, and so far resemble the larger ones of the typhus eruption, or that in some case of typhus rash a few rose spots were not present for a time, and then disappeared. There is no proof, however, that such cases, although surmised, do ever occur. The general fact still remains, that a well-marked rose rash belongs to fever having peculiar symptoms, and that an equally well-marked mulberry rash belongs to another variety of fever, having its symptoms, one of the most striking of which is (as the above list shows) the diarrhœa and diseased intestine in the former, and the perfectly healthy intestine, in those who were examined after death, in the latter. With regard to the duration of the eruption in each variety, it was found that the mulberry rash persisted throughout the disease, and that the rose rash constantly faded and reappeared in a succession of crops. I think Dr. Jenner mentioned four days as the duration of a single macule, and I believe the spots usually begin to disappear about this time, but I have found it not unusual for them to remain six or seven days. There are two other facts worthy of remembrance in the history of the rose rash: the one is, the occasional reappearance of the eruption during a relapse, and the other has reference to a difficulty which sometimes occurs in judging of the nature of the rash which is about to appear at the onset of the fever; for, previous to the development at the perfect form, a general pink efforescence of the skin may exist, and be easily mistaken for the incipient stage of the mulberry rash. In conclusion, then, we must say, with respects to the eruptions, that two well-marked varieties characterize two forms of fever, but that in some exceptional cases they are for a time with difficulty distinguishable."

Afterwards, Dr. Wilks speaks of that condition of the intestine which constitutes the main distinction between the fevers in question.

"We have seen that in all those cases where the rose rash existed, diarrhœa was also present, besides the other well-known symptoms of intestinal disease—as the full doughy abdomen, the flushed cheek, the red tongue, &c. It will be seen, also, that diarrhœa is mentioned as having occurred in some cases where the mulberry rash existed; but it must also be observed, at the same time, that no concomitant symptoms of bowel affection were present, and that in the fatal cases, with this eruption, there never was the slightest morbid appearance found in the ileum. In perusing the case of typhus where diarrhœa is mentioned, the observations which I made in my preliminary remarks must be remembered, that a loose evacuation is often to be anticipated at the termination of the fever, for I related cases where this had occurred, and no disease of the intestine had been found, and stated that, on the post-mortem table, we see the contents of the bowels in typhus fever to be fluid. Whether the evacuation occurs as a crisis, I will not undertake to say; but I can state that it is by no means uncommon at the expiration of the fever, and when the mulberry rash is fading, for one or two large liquid fecal evacuations to take place. This symptom cannot, and ought not, then, to be constituted into a diarrhœa, much less to be taken as evidence of a diseased bowel. Notwithstanding, as I have myself witnessed, a difficulty has arisen wiih respect to this symptom when a patient has been seen during its occurrence for the first time. In two cases of typhus in my list, diarrhœa is said to exist at the commencement of the disease; but as both patients had taken aperient medicine before admission, the symptoms might justly be attributed to this fact. If, however, a little diarrhœa did take place occasionally in a case of typhus, it is no more than might be expected with such a condition of fluids as is supposed to exist in this disease, for its occurrence, we know, is very common in pyæmia and analogous affections, where the blood is in a dyscrasic state. In such diseases we do not look to this symptom alone as evidence of a diseased bowel, unless it be constant, and other reasons for suspecting its existence be present; but rather we consider it as one of the means which nature employs to carry off the poisonous matter from the blood. We must say, then, that a patient with typhus having occasionally a loose evacuation, presents a symptom which might be expected, and cannot be taken as evidence of a diseased bowel. As a rule, however, it seldom occurs during the progress of fever, but not unfrequently at the close; and in most cases after death the intestinal contents are found fluid. In the sixth case, of a man who had typhus, and no diarrhœa, the intestines were found full of liquid fecal matter; and therefore, if this man had lived but a short time longer, he must have had a large liquid evacuation. Some years ago I witnessed the following case: A woman, æt. 48, was very ill with typhus fever, and covered with a mulberry rash, of which some of the spots at the end of a fortnight had become petechial. A castor-oil injection was then administered, a copious fluid evacuation followed, and continued until death, a few hours afterwards. This was apparently a case of typhus, dying of diarrhœa. The intestine, however, was found quite healthy. How far the treatment produced an over-action of the bowel, which increased the exhaustion and assisted in killing the patient, is questionable."

The cases related in this report also show very clearly the very marked difference which there is in the natural history of these two fevers; and this difference is well put by Dr. Wilks, together with some practical bearings of the difference, which we cannot refrain from quoting.

"If, as we shall find, these forms of disease pursue a certain definite course of their own, it is evident it must be of the very highest importance to have an accurate knowledge of their duration, and, therefore, in any individual case about which we may be consulted, it will be our first duty to ascertain the exact date of the illness; for not only will this knowledge be necessary to form a prognosis, but upon it will depend in a great measure the success of the treatment. In looking at the cases of typhus, it will be seen that on the third or fourth day of the disease, the patient is exceedingly ill; his nervous and muscular powers are quite prostrated; he is, perhaps, delirious; and at that early period his skin is covered with rash. Now, in case of typhoid, the early progress of the case is much slower. The patient, whom we may suppose to be taken ill at the same time as the typhus one, is still at his employment, while the latter is ill in bed, and does not seek admission until about the tenth day. He is then becoming very ill, and a rash is appearing. If he present himself earlier, as at the end of seven days, he walks to the hospital, and no appearance of eruption has yet been discovered; he still preserves his intelligence, and is able to give a complete history of his symptoms. These, at the onset, are insiduous, as they are often at first not more urgent than those which attach themselves to a common cold—a shivering, loss of appetite, slight headache, &c.—sometimes an epistaxis. In typhus, on the other hand, the symptoms are at an early period of a much more violent character, the headache is often intense, and the depression very great, with an extreme aching of the limbs. The same rapidity of symptoms characterizes typhus throughout its subsequent career; the subject of it being exceedingly ill on the fourth day, sinks lower and lower until between the twelfth and fourteenth day, when he begins to improve. The time of duration of the disease in all probability is uniform, but the want of exactness met with in this respect is due to the difficulty of ascertaining accurately the first onset of the symptoms. As a rule, the fever comes to an end on the thirteenth or fourteenth day. The typhoid, on the other hand, runs a much longer course. The patient with this disease is ill at least three weeks before any sign of recovery takes place, &c. The time of this occurrence is not so definite as in typhus, and which want of precision is due to the same cause—the difficulty of ascertaining the particular day on which the symptoms commenced. It is never until the expiration of three weeks, however, that a change is observed, and this is generally seen to happen on some day during the fourth week. As I before said, I was hardly prepared for this uniformity in the course of the disease; but it affords an explanation of a fact which is constantly heard spoken of in reference to fever—the rapid progress of a case under one treatment, and the lingering nature of the illness under another. It will be seen by an observation of the above cases, that whether the patients entered the hospital early or late, the disease ran a certain course. Those which came in towards the close of the fever soon got better, and those which came in early, daily got worse, and this was seen to occur under all kinds of treatment. Some simply had salines administered, some had tonics, and others stimulants from the beginning, but it was all the same, nothing stayed the progress of the disease. I exclude in this statement the plan of Dr. Dundas, by large doses of quinine, of which I have little or no experience; and of course, in speaking of the duration of fever, I do not refer to the subsequent effects of the disease—as pulmonary or abdominal phthisis, *&c.*—which may delay the ulterior recovery to an indefinite period."

This report does not throw much light upon one point—and that is, as to whether typhus and typhoid are ever intercommunicable; nor was this to be expected in hospital practice. There was not a tittle of evidence, however, in favor of intercommunicability. The post-mortem disclosures presents no novelty. They agree fully with those already known.

In a word, the evidence contained in the report is irresistible; and Dr. Wilks is quite warranted in saying, as he does say—

"I say, then, as regards the typhus and typhoid genera, that in the majority of instances the two are plainly distinguishable, and that in few cases in which some doubt may arise, on account of the obscurity of a rash or some other symptom, a light will subsequently be thrown upon them by the completion of the history, and it will then be clearly seen to which type they belong; and if there be yet one or two cases (in any given period) concerning which an obscurity hangs throughout the whole duration of the illness, they will probably be fewer in number than those instances in which an equal doubt may exist as to the presence of fever or a local inflammatory disease of the head or chest. From such exceptional cases, therefore, it were no more just to confound the two species, than to say fever, pneumonia, and arachnitis were identical, because in some few instances it were not possible to distinguish these latter apart. I believe, then, myself, that the connection between the two kinds of fever is no greater than exists between many other forms of disease; but the greater tendency to confound them has been owing to the want of a correct knowledge of their history, as well as to the non-recognition of some particular and important symptoms attending them."—*Ranking's Abstract.*

